# Cell states associated with amyloid pathology and cognitive resilience in living human brain

**DOI:** 10.1002/alz70855_097567

**Published:** 2025-12-23

**Authors:** Evan Macosko

**Affiliations:** ^1^ Broad Institute of MIT and Harvard, Cambridge, MA, USA

## Abstract

**Background:**

The identification of cellular and molecular phenotypes specifically associated with Alzheimer's disease (AD) pathology in human brain tissue primarily relies upon postmortem tissue specimens. Limitations of these specimens include: 1) they tend to derive from older patients at later stages of disease progression; 2) cellular states can be affected by processes occurring peri‐ and post‐mortem; and 3) cognitive trajectories cannot be ascertained after tissue acquisition. Normal pressure hydrocephalus (NPH) is a disease in which older patients often require shunt placement through the frontal cortex to relieve excessive cerebrospinal fluid (CSF) buildup. Approximately 30% of patients who undergo this procedure have amyloid and/or tau pathology within biopsied samples of their frontal cortex, providing an opportunity to directly measure pathology‐associated cellular states in living human brain specimens.

**Methods:**

Single‐nucleus RNA sequencing (snRNA‐seq) was performed on frontal cortex biopsies from 294 NPH patients (141 controls, 104 amyloid‐positive, 36 amyloid and tau‐positive, 13 tau‐only positive). These profiles were integrated with cortical snRNA‐seq data from 885 postmortem AD case‐control subjects, creating a dataset of 10 million cell profiles. This large‐scale analysis enabled the identification of cellular substates linked to early and late pathology. eQTL and mediation analyses pinpointed cellular sites of action for AD‐associated genetic loci. Additionally, cognitive trajectories of NPH donors were tracked for 3–5 years post‐biopsy, allowing identification of cell states linked to resilience or vulnerability to amyloid‐related cognitive decline.

**Results:**

Amyloid and tau pathologies induced distinct cellular perturbations, including changes in neuronal and glial proportions. In microglia, amyloid exposure expanded GPNMB‐ and LPL‐expressing substates enriched for AD‐GWAS genes. Mediation analyses showed that most risk genes, like *BIN1* and *PICALM*, acted broadly across microglial substates, while others, such as *APOC1* and *GRN*, were specific to certain states. eQTL effects revealed shared regulatory mechanisms and unique amyloid‐specific populations. Cognitive analyses identified molecular markers linked to resilience and vulnerability over time.

**Conclusion:**

NPH biopsies provide unique insights into cellular states associated with amyloid pathology and cognitive resilience. Integrating snRNA‐seq from living and postmortem tissue bridges knowledge gaps in AD research, highlighting molecular and cellular changes underlying disease progression and resistance.

